# Crimean-Congo Hemorrhagic Fever Virus in Humans and Livestock, Pakistan, 2015–2017

**DOI:** 10.3201/eid2604.191154

**Published:** 2020-04

**Authors:** Ali Zohaib, Muhammad Saqib, Muhammad A. Athar, Muhammad H. Hussain, Awais-ur-Rahman Sial, Muhammad H. Tayyab, Murrafa Batool, Halima Sadia, Zeeshan Taj, Usman Tahir, Muhammad Y. Jakhrani, Jawad Tayyab, Muhammad A. Kakar, Muhammad F. Shahid, Tahir Yaqub, Jingyuan Zhang, Qiaoli Wu, Fei Deng, Victor M. Corman, Shu Shen, Iahtasham Khan, Zheng-Li Shi

**Affiliations:** National University of Sciences & Technology, Islamabad, Pakistan (A. Zohaib);; Wuhan Institute of Virology of the Chinese Academy of Sciences, Wuhan, China (A. Zohaib, J. Zhang, Q. Wu, F. Deng, S. Shen, Z.-L. Shi);; University of Agriculture, Faisalabad, Pakistan (M. Saqib, M.H. Tayyab, M. Batool);; University of Karachi, Karachi, Pakistan (M.A. Athar); Ministry of Agriculture and Fisheries, Muscat, Oman (M.H. Hussain);; PMAS Aird Agriculture University, Rawalpindi, Pakistan (A. Sial, J. Tayyab);; Livestock and Dairy Development Department, Punjab, Pakistan (H. Sadia, U. Tahir);; Government College University Faisalabad, Faisalabad (Z. Taj);; Shaheed Benazir Bhutto University of Veterinary and Animal Sciences Sakrand, Sindh, Pakistan (M.Y. Jakhrani);; Livestock & Dairy Development Department Balochistan, Quetta, Pakistan (M.A. Kakar);; University of Veterinary and Animal Sciences, Lahore, Pakistan (M.F. Shahid, T. Yaqub, I. Khan);; National Virus Resource Center, Wuhan (Q. Wu, F. Deng, S. Shen);; Humboldt-University and Berlin Institute of Health, Berlin, Germany (V.M. Corman);; German Centre for Infection Research, Berlin (V.M. Corman)

**Keywords:** Crimean-Congo hemorrhagic fever virus, Pakistan, human, livestock, viruses, ticks, parasites, vector-borne infections, Crimean-Congo hemorrhagic fever, CCHF, CCHFV, zoonoses, *Suggested citation for this article*: Zohaib A, Saqib M, Athar MA, Hussain MH, Sial A, Tayyab MH, et al. Crimean-Congo hemorrhagic fever virus in humans and livestock, Pakistan, 2015–2017. Emerg Infect Dis. 2020 Apr [*date cited*]. https://doi.org/10.3201/eid2604.191154

## Abstract

We detected Crimean-Congo hemorrhagic fever virus infections in 4 provinces of Pakistan during 2017–2018. Overall, seroprevalence was 2.7% in humans and 36.2% in domestic livestock. Antibody prevalence in humans was highest in rural areas, where increased contact with animals is likely.

Crimean-Congo hemorrhagic fever (CCHF) is caused by CCHF virus (CCHFV), an emerging zoonotic virus belonging to the order Bunyavirales within the family *Nairoviridae.* The virus is maintained through a tick–vertebrate transmission cycle (*1*); the primary vectors are ticks from the genus *Hyalomma* (*2*,*3*). Wild and domestic mammals, including livestock species such as sheep, goats, and cattle, are amplifying hosts (*2*). CCHFV is listed as a high-priority zoonotic pathogen of humans in the World Health Organization Research and Development Blueprint (https://www.who.int/blueprint/priority-diseases) because of its potential to cause a public health emergency and the absence of specific treatment and vaccines.

Most human infections occur through the bite of infected ticks. Blood and other bodily fluids of infected animals represent an additional source for human infections. In humans, CCHF is manifested by fever, headache, vomiting, diarrhea, and muscular pain; bleeding diathesis with multiorgan dysfunction is seen in severe cases (*4*–*6*). CCHFV is endemic over a wide geographic area, spanning from western Asia to southern Europe and over most of Africa (*2*). Since the earliest identified CCHF case in 1976 (*7*), several outbreaks of CCHFV infection have been reported from Pakistan. Although Pakistan has the fourth highest number of human cases in Asia (*2*), no comprehensive surveillance study has been conducted to determine the disease prevalence in human and animal populations of Pakistan. Therefore, we determined the countrywide risk for CCHFV infection by detecting the virus and antibodies in livestock, ticks, and humans.

## The Study

During 2017–2018, we tested 3,710 serum samples from 1,872 humans and 1,838 domestic animals (311 buffaloes, 480 camels, 183 cattle, 440 goats, and 424 sheep) for antibodies against CCHFV (Appendix). We also screened 98 blood plasma samples (24 from goats, 28 from buffalo, and 46 from cows) and 774 ticks (509 *Hyalomma* spp., 134 *Rhipicephalus* spp., 77 *Haemaphysalis* spp., and 54 *Rhipicephalus* [*Boophilus*] spp.), sampled from livestock in Punjab Province, for CCHFV antigen by commercial ELISA (VectoCrimea-CHF-antigen ELISA; Vector-Best, https://vector-best.ru).

**Figure 1:**
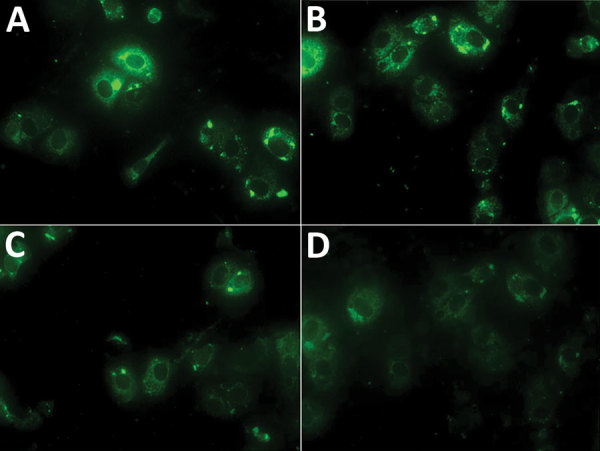
Indirect immunofluorescence assay results for Crimean-Congo hemorrhagic fever virus for 4 samples from humans that were positive by ELISA, Pakistan, 2016–2017. A, B) Samples at 1:100 dilution. C, D) Samples at 1:20 dilution.

We found a total of 51 (2.7%) human samples to be positive for CCHF antibodies by using a 2-step approach, ELISA and confirmatory testing by immunofluorescence assay (Figure 1; Appendix). We observed significantly higher than average prevalence (p<0.01) among samples from Balochistan (5.7%, 95% CI 3.4%–9.3%) and the lowest prevalence among those from Sindh (1.1%, 95% CI 0.5%–2.3%). Samples from Balochistan were almost 6 times (odds ratio [OR] 5.6, CI 2.0–18.0) more likely to test positive than those from Sindh. Seroprevalence increased uniformly with age; we saw the highest level of CCHFV antibodies in persons >65 years of age (Table 1). Of the 51 positive samples, 28 (2.7%, 95% CI 1.8%–3.8%) were from female and 23 (2.8%, 95% CI 1.9%–4.2%) from male participants. We observed significantly higher (p<0.01) seroprevalence among livestock farmers (3.2%, 95% CI 2.4%–4.2%) compared with the general population (0.6%, 95% CI 0.1%–2.3%).

**Table 1:** Univariate analyses of 1,872 human samples positive for Crimean-Congo hemorrhagic fever virus by ELISA, Pakistan, 2017–2018

Category	No. positive/no. tested	Prevalence, % (95% CI)	Odds ratio (95% CI)	p value
Province				<0.001
Punjab	25/930	2.7 (1.8–4.0)	2.6 (1.0–7.7)	
Khyber Pakhtunkhwa	6/128	4.7 (2.1–10.0)	4.6 (1.2–17.5)	
Balochistan	14/247	5.7 (3.4–9.3)	5.6 (2.0–18.0)	
Sindh	6/567	1.1 (0.5–2.3)	1.0	
Age, y				0.451
15–24	7/438	1.6 (0.8–3.3)	1.0	
25–34	19/730	2.6 (1.7–4.0)	1.6 (0.7–4.7)	
35–44	12/388	3.1 (1.8–5.4)	2.0 (0.7–6.0)	
45–54	9/226	4.0 (2.1–7.5)	2.6 (0.8–8.2)	
55–64	3/70	4.3 (1.4–12.5)	2.8 (0.5–12.4)	
>65	1/20	5.0 (0.7–28.2)	3.2 (0.1–27.3)	
Sex				0.832
F	28/1,055	2.7 (1.8–3.8)	1.0	
M	23/817	2.8 (1.9–4.2)	1.1 (0.6–1.9)	
Occupation				0.006
Livestock farmer	49/1,523	3.2 (2.4–4.2)	5.8 (1.5–49.2)	
General population	2/349	0.6 (0.1–2.3)	1.0	

Of the 1,838 animals, 666 (36.2%) were positive for CCHF by a commercial ELISA (ID Vet, https://www.id-vet.com). The prevalence of CCHFV antibodies was significantly higher (p<0.01) among camels (56.7%, 95% CI 52.1%–61.2%) than among cattle (44.3%, 95% CI 36.9%–51.8%), sheep (32.6%, 95% CI 28.1%–37.2%), buffalo (29.6%, 95% CI 24.6%–35%), and goats (18.9%, 95% CI 15.3%–22.8%) (Appendix Tables 1–5). Camels were almost 6 times (OR 5.6) more likely to be positive than other species. As we found for humans, we found significantly higher (p<0.01) seroprevalence of CCHFV antibodies among animals from Balochistan (59.3%, 95% CI 54.1%–64.5%) than among animals from the other regions tested (Table 2).

**Table 2:** Univariate analyses of 1,838 livestock samples positive for Crimean-Congo hemorrhagic fever virus by ELISA, Pakistan, 2017–2018

Category	No. positive/no. tested	Prevalence, % (95% CI)	Odds ratio (95% CI)	p value
Species				<0.001
Camel	272/480	56.7 (52.1–61.2)	5.6 (4.2–7.6)	
Cattle	81/183	44.3 (36.9–51.8)	3.4 (2.3–5.0)	
Sheep	138/424	32.6 (28.1–37.2)	2.1 (1.5–2.8)	
Buffalo	92/311	29.6 (24.6–35.0)	1.8 (1.3–2.5)	
Goat	83/440	18.9 (15.3–22.8)	1.0	
Province				<0.001
Balochistan	213/359	59.3 (54.1–64.5)	7.6 (5.4–10.6)	
Khyber Pakhtunkhwa	230/439	52.4 (47.6–57.1)	5.7 (4.1–7.9)	
Punjab	159/644	24.7 (21.4–28.2)	1.7 (1.2–2.40)	
Sindh	64/396	16.2 (12.7–20.2)	1.0	
Sex				0.377
F	552/1,504	36.7 (34.3–39.2)	1.1 (0.9–1.4)	
M	114/334	34.1 (29.1–39.5)	1.0	
Age, y				<0.001
<5	332/1,121	29.6 (27–32.4)	1.0	
>5	334/717	46.6 (42.9–50.3)	2.1 (1.7–2.5)	

We built a binary logistic regression model to evaluate possible risk factors for CCHFV seropositivity in animals and humans. The final model (Appendix Table 6) at the animal level indicated that the animals with highest risk for being antibody positive are livestock from Balochistan (OR 12.1, 95% CI 7.7–19.1), buffalo (OR 4.4, 95% CI 2.8–6.8), and animals >5 years of age (95% OR 1.3, CI 1.0–1.7). However, the NR^2^ value of 0.277 and the Hosmer-Lemeshow goodness-of-fit test (χ^2^ 22.005; p = 0.003) indicated that this is a poor model for predicting CCHFV exposure in the sampled livestock population.

In humans, we found the chance of exposure to CCHFV was highest for populations from Balochistan (OR 6.6, 95% CI 2.5–17.5) (Appendix Table 7) and in persons belonging to the herdsman profession (OR 7.3, CI 1.7–30.2). The values of NR^2^ (0.070) and Hosmer-Lemeshow goodness-of-fit test (χ^2^ 1.490; p = 0.684) indicated that our model is a reasonable model for predicting past exposure to CCHFV in the tested human population.

**Figure 2:**
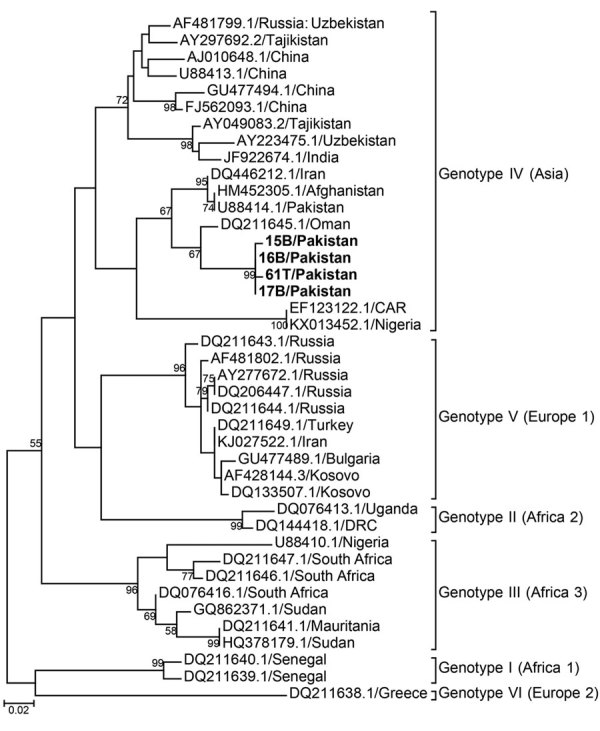
Phylogeny of Crimean-Congo hemorrhagic fever virus, Pakistan, 2016–2017 (bold text), and reference viruses, based on partial small gene sequences. Numbers at branch nodes indicate bootstrap support values. GenBank accession numbers are provided for reference sequences. Scale bar indicates nucleotide substitutions per site.

Four plasma samples from buffalo and 4 *Rhipicephalus* tick samples tested positive for CCHFV antigen by ELISA. Of these 8 positive samples, we confirmed 1 tick (T61) and 3 buffalo samples (15B, 16B, and 17B) through partial amplification and sequencing of the small (S) segment (260 bp). The 4 partial sequences of CCHFV S segments (GenBank accession nos. MN135938–MN135941) were 97%–95% identical to virus sequences found in Afghanistan (accession no. JX908640.1), Iran (accession no. KX096702.1), and Oman (accession no. KY362516.1) and clustered together with genotype IV (Asia) (Figure 2). We obtained full-length sequences of the CCHFV S, medium (M), and large (L) segments (accession nos. MN135942–MN135944) from the tick sample by sequencing on a HiSeq3000 (Illumina, https://www.illumina.com). Phylogenetic trees for the S, M, and L segments showed that the T61 strain clustered with genotype IV (Asia) (Appendix Figures 1–3).

## Conclusions

This countrywide study of CCHFV in Pakistan strongly suggests virus circulation in specific geographic regions and suggests CCHFV foci and a potential source of human infections. Detection of the antibodies in domestic livestock species (including sheep, goats, cattle, buffalo, and camels) indicates a potential role of these animals in human infections. Demonstration of the virus in animal blood plasma and tick samples by reverse transcription PCR provides strong evidence of active circulation of CCHFV in Pakistan. Furthermore, genetic characterization of the virus reconfirms the circulation of genotype IV in Pakistan (*8*). Of interest, we found no *Hyalomma* tick positive for CCHFV; CCHFV has been reported from *Rhipicephalus* ticks from Iran and clustered together with strains from Pakistan and Iran, indicating that *Rhipicephalus* ticks have been naturally infected with closely related virus in the region (*9*). Our study further confirms the role of *Rhipicephalus* ticks in CCHFV circulation in the region. We observed higher prevalence of CCHFV antibodies in camels than in animals of other species, indicating the importance of camels in CCHFV ecology in Pakistan.

A high proportion of seropositive humans from Balochistan and Khyber Pakhtunkhwa with a history of exposure to animals is in concordance with earlier reports of CCHF in humans from these areas. The rural economy of Balochistan and Khyber Pakhtunkhwa is based on livestock production, and the increased contact with animals may explain the higher antibody prevalence in humans from these areas. Furthermore, the prevalence of antibodies was significantly higher among herdsmen than among the general population.

In summary, our results indicate the ongoing circulation of CCHFV among animals and humans in some regions of Pakistan. Longitudinal surveys to identify and define the genomic diversity of CCHFV in Pakistan and investigations to explore the exact role of camels in the ecology of this virus would help clarify the risk to the general population and occupational hazards for livestock farmers and veterinarians.

Appendix Additional information about surveillance of tickborne Crimean-Congo hemorrhagic fever virus in humans and livestock, Pakistan, 2016–2017.
