# Supplementary material for: Crimean-Congo Hemorrhagic Fever Virus in Humans and Livestock, Pakistan, 2015–2017
Source: Emerg Infect Dis. 2020 Apr;26(4):773–7. doi: 10.3201/eid2604.191154 (PMC7101105; doi:10.3201/eid2604.191154)
Supplement: Appendix — Additional information about surveillance of tickborne Crimean-Congo hemorrhagic fever virus in humans and livestock, Pakistan, 2016–2017. [file 19-1154-Techapp-s1.pdf]

Article DOI: https://doi.org/10.3201/eid2604.191154

Crimean-Congo Hemorrhagic Fever Virus in Humans and Livestock, Pakistan

Appendix

Human Samples

Human samples used in this study were part of the samples collected randomly during a countrywide arbovirus surveillance study conducted under the International Cooperation on Key Technologies of Biosafety along the China-Pakistan economic corridor. Due to limited availability of resources, troublesome geopolitical status, and difficulty in convincing farmers to provide samples, convenience sampling was performed. Because seroprevalence of the disease under study was unknown in these areas of Pakistan, the sample size was calculated by considering the expected prevalence to be 50% with confidence limits of 95% and desired absolute precision of 5% to take maximum numbers of samples (*1*). We collected a total of 1,872 human serum samples during 2017–2018. We also recorded information on individual factors such as demographics, animal ownership, and education status. In total, we collected 930 (49.68%) samples from Punjab, 567 (30.29%) from Sindh, 247 (13.19%) from Balochistan, and 128 (6.84%) from Khyber Pakhtunkhwa. Of 1,872 samples, 1,055 (56.36%) were from female and 817 (43.64%) from male participants. Humans sampled in this study were divided into 6 age groups: 15–24 years (n = 438), 25–34 years (n = 730), 35–44 years (n = 388), 45–54 years (n = 226), 55–64 years (n = 70) and ≥65 years (n = 20). Mean age of the sampled population was 33.36 years.

A clinician or phlebotomist collected 4 mL of blood from consenting study participants from the peripheral vein into the gel-clot activator containing vacutainer (Improvacuter, Germany). The samples were shipped to the University of Agriculture, Faisalabad, Pakistan, while maintaining the cold chain. Samples were then centrifuged at 5,000 rpm for 12 minutes; then serum was harvested and aliquoted in cryovials (Imec,China) and preserved at –40°C until subjected to further experimentation.

Animal and Tick Samples

For animal sampling, we selected 14 districts from Punjab, 3 from Khyber Pakhtunkhwa, 7 from Balochistan, and 5 from Sindh. We chose sampling sites on the basis of animal populations, ease of sampling, and the presence of veterinary clinics. We sampled a total of 311 buffaloes, 480 camels, 183 cattle, 440 goats, and 424 sheep; from each animal, we drew ≈4 mL blood aseptically directly into the gel-clot activator containing vacutainer (Improvacuter). We centrifuged samples, and then harvested serum samples and stored them at −40°C. The animal sampling period spanned 30 months during 2015–2018. In addition, Punjab Livestock and Dairy Development Department provided 98 plasma samples (24 from goats, 28 from buffaloes, and 46 from cows) from the household livestock of the CCHF-suspected human case-patients.

We collected a total of 509 *Hyalomma* spp., 134 *Rhipicephalus* spp., 77 *Haemaphysalis* spp., and 54 *Rhipicephalus* spp. ticks from livestock from Punjab province of Pakistan. The ticks were collected at the time of field sampling. Animals randomly selected for serum collection were also carefully examined for the presence of ticks; if infested, we collected the ticks by removing them with forceps from the animals. We stored ticks at 4°C in the field and sent them to University of Agriculture Faisalabad, Pakistan, where they were stored at −40°C.

Serologic Testing

For human serum samples, we used a 2-step approach. In the first step, we screened all human serum samples using a commercial ELISA (Vector-Best, https://vector-best.ru) according to the manufacturer's instructions. In the second step, all ELISA-positive serum samples were confirmed by indirect immunofluorescence assay. The cells were fixed with phosphate-buffered saline (PBS, pH = 7.5) containing 4% paraformaldehyde, permeablised with 0.2% TritonX-100, and blocked with 5% bovine serum albumin at 37°C for 1 hour. We added serum samples diluted in 1% bovine serum albumin and incubated plates overnight at 4°C. We washed the culture plates and added FITC-tagged anti-human IgG at a dilution of 1:1,000 (Sigma, USA). We incubated plates for 1 hour, then washed them and added 100 μL of 1× PBS solution. We observed fluorescence using a fluorescence microscope and acquired images. We first tested serum samples at a dilution of 1:100; negative samples were further tested at a dilution of 1:20. We used anti-CCHFV NP monoclonal antibody (43E5) as the positive control.

For animal serum samples, we conducted screening of anti-CCHFV IgGs by ELISA (ID Vet, https://www.id-vet.com). This double antigen multi-species ELISA can detect IgG antibodies against NP protein of CCHFV in caprine, ovine, bovine, and other susceptible species' serum samples.

We used animal plasma samples and ticks to screen CCHFV antigen by VectoCrimea-CHF-antigen (Vector-Best, https://vector-best.ru) ELISA kit according to the manufacturer's instructions.

Reverse Transcription PCR (RT-PCR)

We extracted total RNA from CCHFV antigen–positive samples using TRIzol reagent (Invitrogen, https://www.thermofisher.com) and performed RT-PCR as previously described (*2*). We visualized the PCR product on 0.8%–1% agarose gel after electrophoresis, then performed Sanger sequencing and edited sequences using Geneious R11. We performed BLAST searches using the nonredundant database (https://blast.ncbi.nlm.nih.gov/Blast.cgi). We performed multiple sequence alignment using MAFFT alignment plugin in Geneious R11 (https://www.geneious.com). We performed phylogenetic analysis in MEGA version 7.0 software (https://www.megasoftware.net).

Whole-Genome Sequencing and Analysis

We prepared libraries for next-generation sequencing using Truseq mRNA kit (TruSeq Stranded mRNA Library Prep Kit, Cat # RS-122–2101; Illumina) following the manufacturer's instructions. We performed sequencing on a HiSeq 3000 sequencer (). We analyzed the data in Metavisitor (a suite of galaxy tools) as described previously (*3*). We then used the BLAST guided scaffold to reference align the reads in Geneious R11. We set up RT-PCR reaction to fill the gap in L segment by using 3508 F(CCHF-L): GGCCAGCTTATCACTCATGGA and 3974 R(CCHF-L): CATTCTGCTGCCACCTCCTT primers.

Statistical Analysis

We conducted statistical analysis using R version 3.5.1 (https://www.r-project.org). We investigated positive samples by χ^2 test for the association of important host (location, sex, and

age) risk factors with the occurrence of infection in animals and human populations of Pakistan. We used the analysis packages Epicalc version 2.15.1.0 and DescTools version 0.99.25. For all analyses, $p<0.05$ was considered significant.

We built a binary logistic regression model to evaluate the risk factors for the seroprevalence of CCHFV in animals and humans. We entered all the variables with $p<0.20$ at univariable analysis into the model. We performed backward stepwise removal of the variables with $p>0.05$ until only significant ($p<0.05$) variables remained in the model. We assessed the fit of the final model by values of Nagelkerke R Square (NR^2) and Hosmer and Lemeshow (HL) goodness-of-fit test (*4*).

References

1. Thrusfield M, Christley R. Veterinary epidemiology. 4th edition. Hoboken, NJ: Wiley-Blackwell; 2018.

2. Rodriguez LL, Maupin GO, Ksiazek TG, Rollin PE, Khan AS, Schwarz TF, et al. Molecular investigation of a multisource outbreak of Crimean-Congo hemorrhagic fever in the United Arab Emirates. Am J Trop Med Hyg. 1997;57:512–8. PubMed https://doi.org/10.4269/ajtmh.1997.57.512

3. Carissimo G, van den Beek M, Vernick KD, Antoniewski C. Metavisitor, a suite of galaxy tools for simple and rapid detection and discovery of viruses in deep sequence data. PLoS One. 2017;12:e0168397. PubMed https://doi.org/10.1371/journal.pone.0168397

4. Bursac Z, Gauss CH, Williams DK, Hosmer DW. Purposeful selection of variables in logistic regression. Source Code Biol Med. 2008;3:17. PubMed https://doi.org/10.1186/1751-0473-3-17

Appendix Table 1. ELISA-based prevalence of Crimean-Congo hemorrhagic fever virus in camels, Pakistan

Variable	Category	No. positive/no. tested	Prevalence, % (95% CI)
Province	Punjab	59/184	32.1 (25.7–39.2)
	Khyber Pakhtunkhwa	88/165	53.3 (45.7–60.8)
	Balochistan	125/131	95.4 (90.2–97.9)
Age	<3 y	11/54	20.4 (11.7–33.2)
	3–10 y	221/356	62.1 (56.9–67.0)
	>10 y	40/70	57.1 (45.4–68.2)
Sex	Female	193/281	68.7 (63.0–73.8)
	Male	79/199	39.7 (33.1–46.7)

Appendix Table 2. ELISA-based prevalence of Crimean-Congo hemorrhagic fever virus in cattle, Pakistan

Variable	Category	No. positive/no. tested	Prevalence, % (95% CI)
Province	Punjab	31/117	26.5 (19.3–35.2)
	Balochistan	43/44	97.7 (85.6–99.7)
	Sindh	7/22	31.8 (16.0–53.4)
Age	<3 y	3/17	17.7 (5.8–42.7)
	3–10 y	75/149	50.3 (42.4–58.3)
	>10 y	3/17	17.7 (5.8–42.7)
Sex	Female	77/174	44.3 (37.1–51.7)
	Male	4/9	44.4 (17.7–74.9)

Appendix Table 3. ELISA-based prevalence of Crimean-Congo hemorrhagic fever virus in sheep, Pakistan

Variable	Category	No. positive/no. tested	Prevalence, % (95% CI)
Province	Punjab	11/100	11.0 (6.2–18.8)
	Khyber Pakhtunkhwa	85/182	46.7 (39.6–54.0)
	Balochistan	42/136	30.9 (23.7–39.1)
	Sindh	0/6	0 (0)
Age	<8 mo	13/32	40.6 (25.3–58.1)
	8 mo–4 y	81/261	33.6 (26.0–42.1)
	>4 y	44/131	31.0 (25.7–76.9)
Sex	Female	123/382	32.2 (27.7–37.1)
	Male	15/42	35.7 (22.8–51.1)

Appendix Table 4. ELISA-based prevalence of Crimean-Congo hemorrhagic fever virus in buffaloes, Pakistan

Variable	Category	No. positive/no. tested	Prevalence, % (95% CI)
Province	Punjab	49/123	39.8 (31.6–48.7)
	Sindh	43/188	22.9 (17.4–29.4)
Age	<3 y	8/34	23.5 (12.2–40.5)
	3–10 y	68/243	28.0 (22.7–34.0)
	>10 y	16/34	47.1 (31.2–63.5)
Sex	Female	87/299	29.1 (24.2–34.5)
	Male	5/12	41.7 (18.5–69.2)

Appendix Table 5. ELISA-based prevalence of Crimean-Congo hemorrhagic fever virus in goats, Pakistan

Variable	Category	No. positive/no. tested	Prevalence, % (95% CI)
Province	Punjab	9/120	7.5 (4.0–13.8)
	Khyber Pakhtunkhwa	57/92	62.0 (51.7–71.3)
	Balochistan	3/48	6.3 (2.0–17.7)
	Sindh	14/180	7.8 (4.7–12.7)
Age	<1 y	4/42	9.5 (3.6–22.8)
	1–4 y	70/322	21.7 (17.6–26.6)
	>4 y	9/76	11.8 (6.3–21.2)
Sex	Female	72/368	19.6 (15.8–23.9)
	Male	11/72	15.3 (8.7–25.5)

Appendix Table 6. Final binary logistic regression model to predict the Crimean-Congo hemorrhagic fever virus exposure in the sampled livestock population

Variable	Category	Reference	Odds ratio (95% CI)	p value
Province	Balochistan	Sindh	12.1 (7.7–19.1)	<0.001
	Khyber Pakhtunkhwa	Sindh	10.8 (6.9–16.9)	
	Punjab	Sindh	1.7 (1.1–2.4)	
Species	Camel	Goat	3.3 (2.3–4.7)	<0.001
	Cattle	Goat	4.3 (2.7–6.8)	
	Sheep	Goat	0.9 (0.6–1.3)	
	Buffalo	Goat	4.4 (2.8–6.8)	
Age	>5 y	≤5 y	1.3 (1.0–1.7)	0.048

Appendix Table 7. Final binary logistic regression model to predict the Crimean-Congo hemorrhagic fever virus exposure in the sampled human population

Variable	Category	Reference	Odds Ratio (95% CI)	p value
Province	Balochistan	Sindh	6.6 (2.5–17.5)	0.001
	Khyber Pakhtunkhwa	Sindh	5.7 (1.8–18.1)	
	Punjab	Sindh	2.7 (1.1–6.5)	
Profession	Herdsman	Others	7.3 (1.7–30.2)	0.006

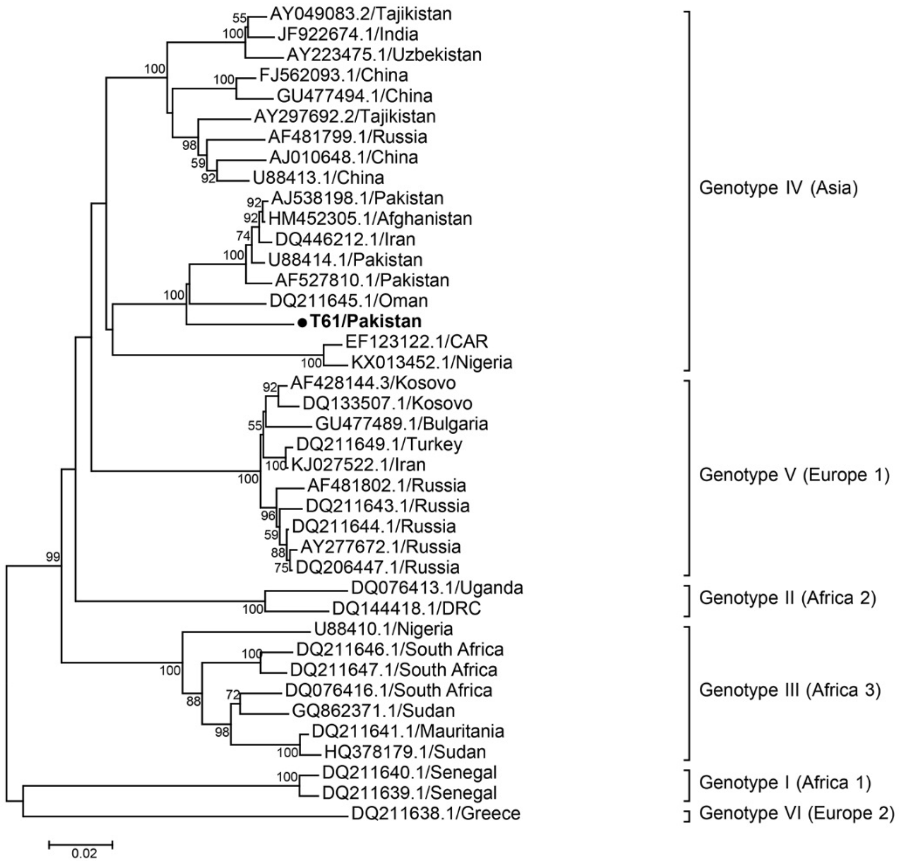

Appendix Figure 1. Phylogenetic analysis of Crimean-Congo hemorrhagic fever virus S segment from Pakistan, compared with reference sequences. Dot and bold text indicate strain detected in this study. Numbers at branch nodes indicate bootstrap support values. The scale bar indicates nucleotide substitutions per site. …

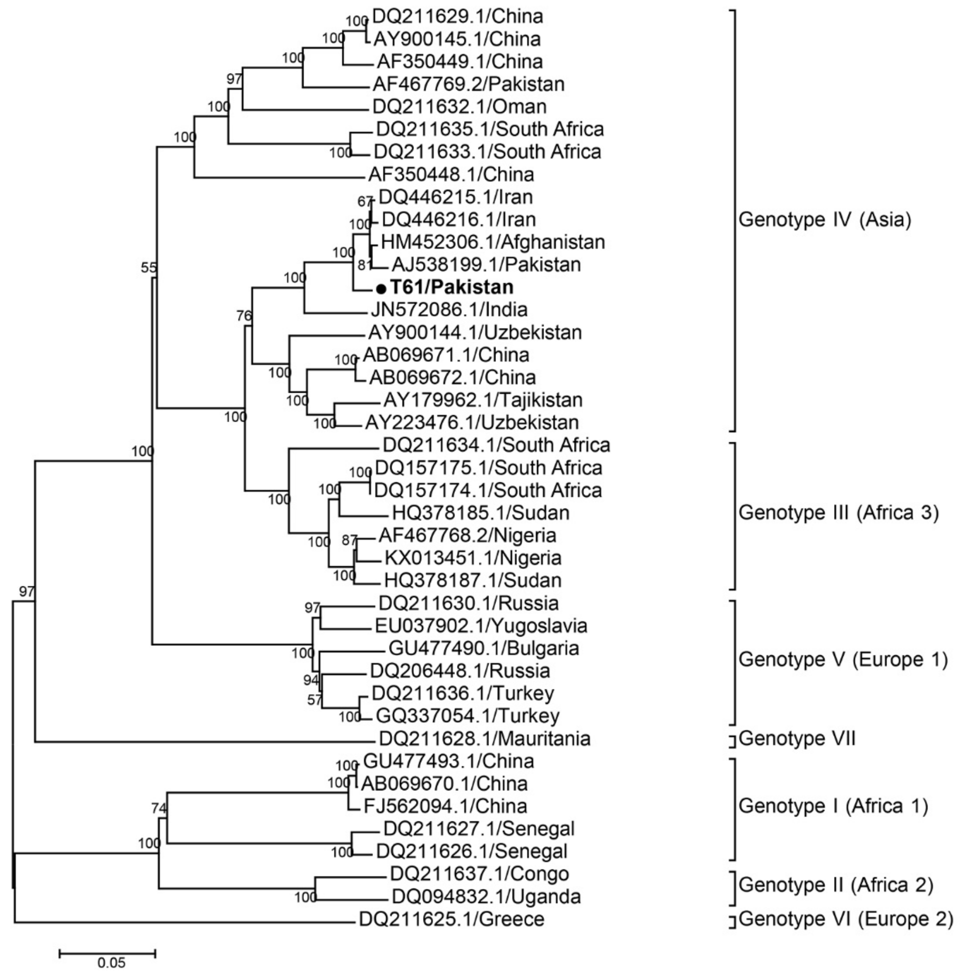

Appendix Figure 2. Phylogenetic analysis of Crimean-Congo hemorrhagic fever virus M segment from Pakistan. Dot and bold text indicate strain detected in this study Numbers at branch nodes indicate bootstrap support values The scale bar indicates nucleotide substitutions per site. …

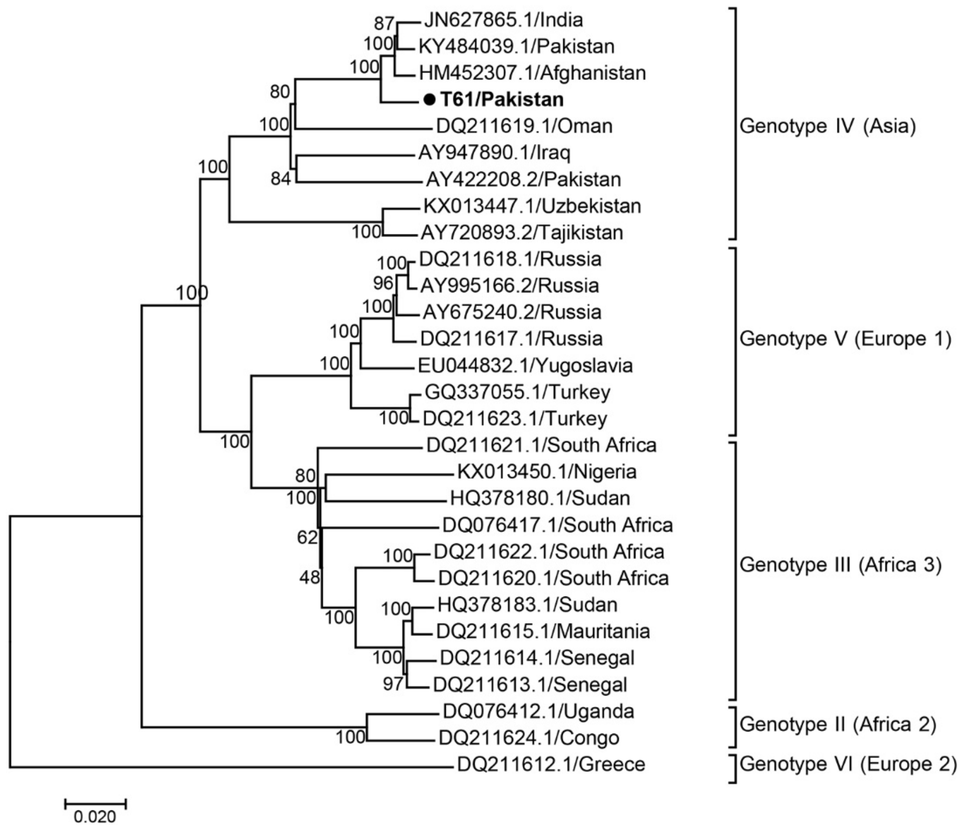

Appendix Figure 3. Phylogenetic analysis of Crimean-Congo hemorrhagic fever virus L segment from Pakistan, compared with reference sequences. Dot and bold text indicate strain detected in this study. Numbers at branch nodes indicate bootstrap support values. The scale bar indicates nucleotide substitutions per site
